# Revenge of the Microbes: How Bacterial Resistance is Undermining the Antibiotic Miracle

**DOI:** 10.3201/eid1110.050738

**Published:** 2005-10

**Authors:** Mike Toner

**Affiliations:** * Atlanta Journal and Constitution

**Keywords:** bacterial resistance, book review, super bugs,

Professional journals these days brim with new developments in the field of antimicrobial resistance, and scarcely a week goes by without a flurry of new reports on "super bugs" in popular media. Given the unrelenting blitz of information, that 2 self-proclaimed "fusty old pedants" could produce a fresh perspective in the ongoing arms race between man and microbe is all the more noteworthy.

Although their traditional milieu is microbiology textbooks, Salyers and Whitt have provided a concise yet readable history of the rise of resistant organisms, as well as the social and economic effect of "these indomitable little critters." The history, from the first hints of penicillin resistance to the recent rise of vancomycin resistance, is as insightful as it is entertaining.

Lay readers will get a digestible dose of the basic science often missing from the mass media. And professionals will find the kind of incisive analysis—and even a touch of humor—that is often missing from scientific journals. Both audiences will find eminently compact descriptions of the major mechanisms that enable bacteria to develop and pass on resistant traits, the hurdles that pharmaceutical companies face in developing new antimicrobial drugs, the dilemmas doctors and patients face in finding better ways to use drugs, and a thoughtful appraisal of possible future trends.

In contrast to prophecies of an approaching "post-antibiotic era," the authors' own "realistic vision of the future" is far from apocalyptic. Still, they worry that increasing numbers of treatment failures like those occurring in hospitals and community settings will erode confidence in the healthcare system. Some diseases, they believe, will remain treatable, some new drugs will emerge, and bacteria, with 3 billion years of evolution on their side, will continue to adapt. So perhaps, they suggest, "the best we can hope for is détente, a running standoff between science and the bugs' remarkable ability to adapt to their changing environment."

